# The antigen presentation function of bone marrow-derived mast cells is spatiotemporally restricted to a subset expressing high levels of cell surface FcεRI and MHC II

**DOI:** 10.1186/1471-2172-11-34

**Published:** 2010-06-30

**Authors:** Jian Gong, Ning-Sun Yang, Michael Croft, I-Chun Weng, Liangwu Sun, Fu-Tong Liu, Swey-Shen Chen

**Affiliations:** 1 Department of Allergy and Immunology, IgE Therapeutics, Inc., San Diego, CA, USA; 2 Agriculture Biotechnology Research Center, Academia Sinica, Taipei, Taiwan; 3 Division of Molecular Immunology, La Jolla Institute for Allergy and Immunology, San Diego, CA, USA; 4 Department of Dermatology, University of California-Davis, Sacramento, CA, USA; 5 Department of Molecular Biology, The Scripps Research Institute, San Diego, CA, USA

## Abstract

**Background:**

At present, it is highly controversial whether pure mast cells can serve as antigen presenting cells, and it is not known whether the capacity of antigen presenting function is temporally restricted to a particular subset of differentiated mast cells. Evidence is presented for a novel surface FcεRI^hi ^, MHC II +, and c-kit + pure mast cell subset, temporally restricted as antigen-presenting cells in the immune axis of T-cell activation.

**Results:**

Bone marrow-derived mast cells (BMMC) cultured in the presence of IL-3 for three weeks are pure mast cells based on surface expression of lineage-specific marker, c-kit and FcεRI. Herein we present the first demonstration that approximately 98.7% c-kit + and FcεRI expressing BMMC, further depleted of any contaminated professional antigen-presenting cells, are still fully capable of presenting antigens, i.e., OVA protein, OVA peptide, and IgE-TNP-OVA, to OVA peptide-specific T-cell hybridomas. Notably, IgE-dependent antigen presentation is more efficient compared to that resulting from direct antigen uptake. Importantly, we present the novel finding that only surface FcεRI^hi ^mast cells, also expressing surface MHC II exhibited antigen-presenting function. In contrast, surface FcεRI^lo ^mast cells without expressing surface MHC II were not capable of antigen presentation. Interestingly, the antigen-presenting function of BMMC was irrevocably lost during the third and fourth week in IL-3 or SCF containing cultures.

**Conclusions:**

This is the first observation to attribute a spatiotemporally restricted antigen-presenting function to a subset of three-week old pure BMMC expressing both high levels of surface FcεRI and surface MHC II. We propose that mast cells play an important role in immune deviating and/or sustaining the activation of infiltrating CD4 T-cells, and modulating T-cell mediated allergic inflammation via its flexibility to present antigens and antigen-IgE complexes.

## Background

Mast cells are traditionally thought of as a secretory cell type releasing inflammatory mediators in IgE-dependent inflammation[1,2]. Their role in innate immunity against pathogens has been uncovered, which is associated with their uptake and clearance of the microbes [3,4]. Mast cells undergo maturation and specialization in different tissue compartments [5-7]. Adoptively transferred progenitors of mast cells from fetal liver and bone marrow repopulate the skin and mucosa of intestine and lung in mast cell-deficient mice as connective-tissue type as well as mucosal type mast cells, distinguished by the granular content and safranin-based staining [5-7].

Although early studies have shown that bone marrow-derived mast cells (BMMC) are capable of presenting bacterial and protein antigens via MHC class I and MHC II pathways [8,9], the purity of mast cells used and whether all BMMC are capable of antigen presentation are not known. Herein, we showed that close to 99% (98.7%) pure, c-kit + BMMC obtained by culturing bone marrow cells in IL-3-containing medium for three-week, followed by removal of putative contaminating antigen-presenting cells (APC), are fully capable of antigen presentation. Herein, we present a novel finding that only the BMMC subset expressing high levels of FcεRI, concomitantly with high levels of MHC II on the cell surface, is capable of antigen presentation, whereas another BMMC subset expressing low levels of surface FcεRI and no detectable cell surface MHC II, fails to present antigens. Moreover, the temporally restricted APC function of BMMC indicates a flexibility of mast cells in playing a role in CD4 T-cell dichotomy and/or sustaining CD4 T-cell activation.

## Methods

### Reagents and chemicals

OVA^323-339 ^(OVAp; ISQAVHAAHAEINEAGR) and pigeon cytochrome c peptide (PCCP; KAERADLIAYLKQATAK) were synthesized on a Rainin Symphony (Peptide Technologies, Tucson, AZ) synthesizer. Rabbit anti-histamine antibody and Texas red-strepavidin were purchased from Sigma (ST. Louis, MO). [^3^H]-thymidine was purchased from Amersham. The T cell hybridoma lines used include: 1) 3Do-54.8, which was provided by Dr. P. Marrack at University of Colorado (Denver, CO) and 2) AD10, restricted to PCC^88-104 ^(PCCP) and IE^k^, which was obtained from Dr. S. Hedrick at University of California at San Diego. A20 (H-2d) and DECK.ICAM (H-2^k^), which were originally transfected with I-E^k ^by Dr. R. Germain, NIH, and further transfected with ICAM-1 by Dr. P. Kuhlman, La Jolla Cancer Research, were employed as APC.

Recombinant GM-CSF and IL-3 were purchased from Genzyme Corp. (Cambridge, MA). TNP-OVA and DNP-specific IgE prepared from IgE-secreting hybridoma, 26.82 were prepared in our laboratory as described [10]. Antigen-IgE complexes were prepared by incubating TNP-OVA and IgE at the same concentrations (wt/wt) for 30 min at room temperature. The following reagents were purchased from BD PharMingen (SD, CA): MAbs anti-B220/CD45R (RA3-6B2), anti-IA^d ^(39-10-8, IgG3/κ), anti-IE^k ^(17-3-3, IgG2a/κ), anti-CD16/32 (2.4G2), anti-CD54 (ICAM-1, 3E2), anti-CD49d (integrin α4, R1-2), anti-CD29 (integrin β1, Ha2/5), anti-CD11a (integrin α_L_, LFA-1 α chain, 2D7), allophycocyanin-conjugated anti-mouse c-kit/CD117 (2B8), FITC-murine control monoclonal IgG1, PE-murine control mIgG1, FITC-rat mIgG1 control and FITC-streptavidin. Anti-CD11c (N418) was obtained from Miltenyi Biotec (Auburn, CA). Hybridoma F4/80 secreting MAb directed against macrophages/monocytes, is a generous gift from Dr. S. Gordon at Oxford University [11]. Hybridomas secreting MAb anti-FcεRI (TW) was obtained from the laboratory of Professor David Holowka at Cornell University (Ithaca, NY). The MAb was previously shown by us to bind to FcεRIα [12]. FITC conjugate of TW was prepared in the laboratory. WEHI-3 constitutively secreting IL-3 was provided by Dr. J. W. Schrader at the University of British Columbia (Vancouver, Canada), and D11 hybridoma secreting IL-3 was provided by Dr. N. Arai at DNAX Research Institute (Palo Alto, CA). Recombinant SCF was obtained from Dr. T. Huff (Virginia Commonwealth Univ, Richmond, VA) [13]. To perform depletion of putative contaminating APC, the reagents were purchased from Miltenyi Biotec (Auburn, CA): anti-B220, and F4/80 anti-macrophage/monocytes, and anti-CD11c (N418 for DC) and mouse anti-rat kappa light chain (MAR18.5) coupled to magnetic MicroBeads™. Procedures were followed according to this vendor.

### BMMC cultures and differential staining

Femurs were collected from three to six-week old female DBA/2 (H-2^d^), BALB/c (H-2^d^) or C3H (H-2^k^) mice (Jackson Lab, Bar Harbor, ME). Marrows were compressed out from the bone cavities with a steady stream of RPMI from a syringe fitted with a 27-gauge needle. Cells were gently spread out and cultured in two 75-flasks in 20% WEHI-3- or D11-conditioned medium in 10% fetal bovine serum (FBS). Non-adherent cells were transferred weekly to a new flask and supplemented with fresh medium. The purity of three-week old mast cell cultures was established by the following assays.

Histamine assay: 5 × 10^4 ^cells were prepared onto a cytospin slide, air dried, fixed in 3% paraformaldehyde for 10 min, washed, and permeabilized in 0.2%Triton X-100/PBS for 2 min. One hundred μl of control rabbit IgG or rabbit anti-histamine at 1:100 dilution (Sigma, ST. Louis, MO) were added and the slide was placed in a moist chamber for 2 hr. Slides were washed and followed by addition of 100 μl of biotinylated oat anti-rabbit IgG at 1:1000 and Texas red-strepavidin at 1:100.

Metachromatic toluidine blue staining: 5 × 10^4 ^BMMC were prepared onto a cytospin slide, air dried, and treated with Mota's fixatives for 10 min, followed by 70% ethanol. Slides were washed with distilled water and treated with acidic toluidine blue solution for 10 min, followed by 66% and 100% ethanol, and finally air-dried and mounted. The magnification was at 200 and 1000, respectively with 20× and 100× objectives

Zymosan ingestion: Less than 1% of cells in BMMC cultures ingested opsonized zymosan particles, after incubation for 1 hr at a zymosan concentration of 1,600 μg/10^6 ^cells, as assessed by Giemsa staining [14], an indication of macrophage contamination.

### Purification of BMMC by magnetic beads

Three-week old BMMC were cultured in 20% WEHI-3-conditioned medium and stimulated with GM-CSF at 100 U/ml with or without supplement of IL-4 at 100 U/ml. About 98-99% of cells were stained positive by toluidine blue, and less than 1-2% of cells were positive by MAbs anti-B220/CD45R (B cells), anti-F4/80 (macrophages/monocytes) or anti-CD11c (DC). Three week old BMMC at 5 × 10^6 ^cells/ml in the bulk cultures of 5 × 10^8 ^cells, were treated with 2.5 μg/ml anti-B220 and anti-F4/80 followed by mouse anti-rat kappa light chain (MAR18.5) coupled to magnetic MicroBeads™ (Miltenyi Biotec) and then passed through anti-CD11c coupled directly to magnetic beads. BMMC undergoing two negative selections were considered pure mast cells. Columns were thoroughly washed, and the adherent cells bound to the beads were harvested.

### BMMC and APC/T cell hybridoma cocultures

To employ as a source of APC, BMMC were primed with recombinant GM-CSF (100 U/ml) overnight prior to harvest. In some experiments, BMMC were then negatively selected by passing through affinity antibody-coupled magnetic beads as described above, and the optimal ratios of BMMC to T-cells were determined to be from 10^2 ^to 10^5^. Routinely, 1 × 10^5 ^BMMC were incubated with 1 × 10^5 ^T cell hybridoma cells (restricted for OVA peptide and I-A^d^) in the presence of IgE-TNP-OVA complexes, TNP-OVA conjugate, native OVA protein or OVA^323-339 ^peptides; or with AD10 T cell line (restricted for PCCP peptide and I-E^k^) in the presence of PCCP for 48 to 72 hr for optimal levels of IL-2 production. Alternatively, BMMC (1 × 10^5 ^cells) were pulsed with the above antigens overnight, washed, and added to 1 × 10^5 ^3Do-54.8 without further antigen addition. Levels of IL-2 secreted by 3Do-54.8, or AD10 were quantified by a bioassay that measures [^3^H]-thymidine incorporation by IL-2-dependent NK-3 cells. Supernatants were routinely harvested after 48 hr of incubation, and IL-2 content was assessed as followed. Thirty μl of supernatant from each sample were added to 10^4 ^IL-2 dependent NK-3 cells for 48 hr. One μCi of ^3^H-thymidine was added to NK-3 cultures overnight before cell harvest. Incorporation of ^3^H-thymidine was determined by a β-counter. Data were expressed as means and standard deviations of CPM (counts per minute) of triplicate or quadruplicate 96 well cultures.

### Sorting by flow cytometry

Cells were harvested from three-week old BMMC cultures. Approximately, 20 × 10^6 ^cells were harvested from three-week old BMMC cultures, resuspended at 10^6^/ml. Cells were incubated at 4°C with 1 μg/ml MAb FITC-anti-FcεRIα (clone TW, rat MAb IgG, a gift of Dr. David Holowka, Cornell Univ) for 45 min, washed and harvested. Flow cytometric sorting experiments were performed using a FACS III cell sorter (Becton-Dickinson, Sunnyvale, CA). An argon ion laser (Model 164-05, Spectra-Physics) was used at a power of 300 mW and an excitation wavelength of 488 nm. FITC (green) fluorescence was detected by a combination of a 520 nm long pass and a 540 nm short pass filter. Sorting was carried out using standard three-droplet deflection criteria with a 70 μm nozzle at the rate of analysis of ~300 cells/s. About 10 × 10^6 ^cells were sorted via one continual operation overnight. Sterilization of the tubing system was performed with 70% ethanol. FcεRI(+) cells were then sorted by flow cytometry by a stringent cut-off point into FcεRI^hi ^(2-3 log intensity) vs FcεRI^lo ^(0-1 log intensity) population. Next, 1 × 10^5 ^FcεRI^hi^/MHCII(+) cells or FcεRI^lo ^/MHCII(-) cells were incubated with 10^5 ^3Do-54.8 T cell hybridomas in the presence of different concentrations of IgE-TNP-OVA, TNP-OVA or IgE (A, B) as well as OVA or OVA peptide (C) for 72 hr. Supernatants were then harvested and levels of IL-2 were tested for [^3^H]-thymidine incorporation by IL-2-dependent NK-3 cells.

### FACS analysis of cell surface and intracellular staining

For viable cell staining, 1 × 10^6 ^cells were resuspended in 100 μl medium containing MAbs at 1-2 μg/ml at 4°C for 30 min, washed, and fixed with 2% paraformaldehyde. For two-color staining for FcεRIα and surface MHC II, cells were incubated with FITC-anti-FcεRIα(TW) and PE-anti-IA^d^. For two-color staining for differentiation of mast cells and basophils, three week old BMMC were stained are incubated with FITC anti-FcεRIα(TW) and PE-anti-mouse c-kit/CD117 (2B8). For intracellular staining, cells were first fixed in 2% paraformaldehyde in Dulbecco-PBS (D-PBS) for 20 min at 4°C. Cells were then washed twice with permeabilization buffer (0.1% saponin in D-PBS without Mg^2+^/Ca^2+^, 1% FBS), and stained cells with FITC- anti-FcεRIα (TW) and PE-anti-IA^d^. FACS analysis was performed on a FACScan (Becton Dickinson). Ten thousand events were collected and analyzed by CELLQuest program (version 1.2.2).

## Results

### Pure BMMC depleted of professional APC present antigens and are more efficient via FcεRI-mediated antigen uptake

**Figure 1:**
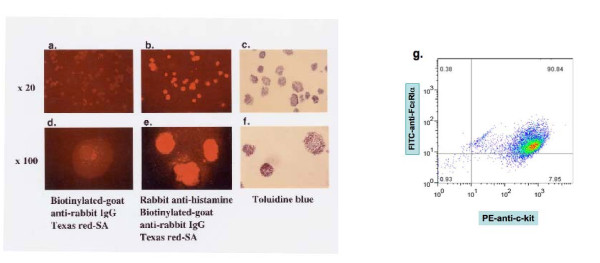
**Characterization of three-week old BMMC cultures**. *Histamine staining: *slides treated with rabbit IgG (Panel a, d) vs rabbit anti-histamine (Panel b, e). *Toluidine blue staining: *5 × 10^4 ^BMMC were prepared onto a cytospin slide, examined respectively with 20× (Panel c) and 100× objectives (Panel f). *Surface FcεRI and c-kit expression: *BMMC were stained with PE-anti-c-kit and FITC-anti-FcεRI analyzed by flow cytometry. Numerically, 98.7% c-kit + cells are mast cells, including c-kit +, FcεRI^hi ^cells (quadrant 3, 90.84%), and c-kit +, FcεRI^lo ^cells (quadrant 2, 7.85%) (g). The procedures were described in Materials and methods.

BMMC cultured in IL-3 for three weeks were shown to present antigen (9). It is critical to ascertain whether BMMC cultures contain pure mast cells without contaminating APC cell types. Nearly all the cells in the cultures were mast cells as ascertained by intracellular histamine staining (Fig. 1: Panels b and e vs a and d). Because macrophages that internalize histamine may also be stained positive [15], the ubiquitous presence of abundant metachromatic granules characteristic of mast cells, was then ascertained by toluidine blue staining (Fig. 1: Panels c and f).

Fig. 1g showed that 98.7% of BMMC (90.84% in the third quadrant plus 7.85% in the second quadrant, Fig. 1: Panel g) exhibited surface receptors for stem cell factor (c-kit, CD117), indicating these cells are of the mast cell and not basophil lineage, and apparently all surface c-kit + BMMC also exhibited surface high affinity IgE Fc receptors delineated by two color FACS (Fig. 1: Panel g). It was previously reported that a minor subset of FcεRI + mast cells, exhibiting surface c-kit, exists in IL-3/SCF containing bone marrow cultures [16]. Thus 0.38% cells (of the fourth quadrant) exhibiting surface FcεRI but not c-kit, may either belong to the putative basophils or to an early lineage mast cells.

Due to the presence of approximately 1% residual cells devoid of mast cell markers, we therefore proceeded to determine whether purified BMMC, negatively selected by adsorption on MACS columns with anti-B220, anti-F4/80 and anti-CD11c, can still present antigens.

**Figure 2:**
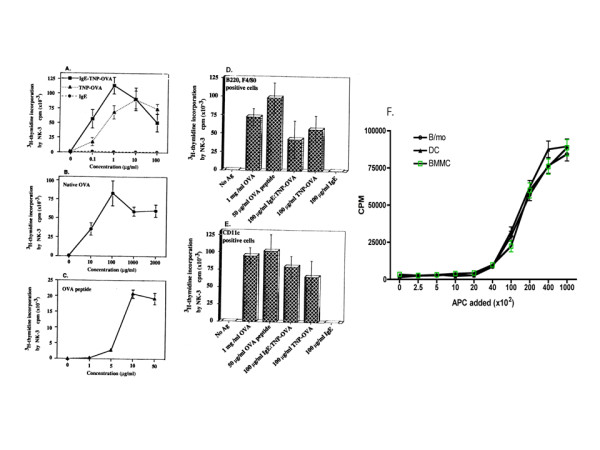
**BMMC cultures depleted of contaminating APC were fully capable of antigen presentation**. 1 × 10^5 ^pure BMMC depleted of B cells/macrophages or DC were incubated for 72 hr with 10^5 ^3Do-54.8 in the presence of IgE-TNP-OVA, TNP-OVA (Panel A), OVA (Panel B) or OVA peptide (Panel C). The residual contaminating APC eluted from the anti-B220/F480 and CD11c MACS columns from bulk cultures (~2 × 10^8 ^cells in order to harvest around 1% contaminating cells for experiments), were prepared. 0.4 × 10^5 ^B cells/macrophages (Panel D) or 0.4 × 10^5 ^DC (Panel E) were incubated for 72 hr with 10^5 ^3Do-54.8 in the presence of IgE-TNP-OVA, TNP-OVA, OVA, or OVA peptide. Next, in the titration experiment, pure mast cells were similarly prepared as above by MACD columns, along with column eluted, residual contaminating APC of these bulk cultures. The different cell types were then incubated with 10^5 ^3Do-54.8 in the presence of OVA peptide, at a APC to T-cell ratios ranged from 1:1 to 0.0025:1 at a serial 2- to 2.5-fold dilutions (Panel F). Levels of IL-2 in 72 hr supernatants were determined by stimulating ^3^H-thymidine incorporation and proliferation of IL-2 dependent NK-3 cells as described in Material and methods.

As shown in Fig. 2A, pure mast cells, depleted of contaminating B-cells, monocytes, and dendritic cells were fully capable of antigen presentation. Mast cells treated with IgE-TNP-OVA complexes were more effective in antigen presentation than those treated with TNP-OVA alone. Approximately four-fold enhancement of ^3^H-thymidine incorporation was observed with mast cells treated with 0.1 μg/ml IgE-TNP-OVA as compared to those treated with similar dose of TNP-OVA. Moreover, close to two-fold enhanced ^3^H-thymidine uptake was observed in mast cells treated with 1 μg/ml IgE-TNP-OVA as compared to those treated with TNP-OVA, while the difference in efficacies of presenting IgE complexes vs haptenated antigen alone, was not noticeable in higher dose range from10 to 100 μg/ml. Purified BMMC were also able to process and present native OVA as well as present OVA peptide to T cells (Fig. 2B and Fig. 2C). On the other hand, mast cells were inefficient in presenting antigens at levels from 0.001 to 0.01 μg/ml (not shown).

Next, to prove the less than 1% contaminating cells were indeed effectively removed, adherent cells, physically separated from pure mast cells, eluted from MACS columns in the same experiment of these bulk cultures, were then examined for APC function. As shown in Fig. 2D and Fig. 2E, B cells/macrophages and dendritic cells were potent in presenting different forms of antigens. And as anticipated, similar efficacy of antigen presentation was observed in these contaminating APC, treated with IgE antigen complexes vs haptenated antigens, indicating FcεRI-mediated antigen focusing did not play a role in these professional cell types. Taken together, these experiments showed that residual contaminating APC exhibiting the respective APC surface markers and APC function, were indeed effectively removed from the three-week BMMC cultures; and that mast cells, even purer than the starting highly pure c-kit + mast cells, were still fully capable of antigen presentation in the absence of contaminating APC.

Next, we evaluated efficacies of antigen presentation at a more extensive titration of mast cells to T-cell ratios as compared to contaminating professional APC, recovered from the MACS columns, on a cell-to-cell basis. As shown in Fig. 2F, significant T-cell responses were stimulated by all three types of cells, B-cells/monocytes, DC and mast cells from a range of 1 × 10^5 ^to 2.5 × 10^2^, added to 1 × 10^5 ^T-cell hybridomas, i.e., at APC to T-cell ratios from 1:1 to 0.0025:1. Quantitative CPM reduction was proportional to the number of input APC and T-cell ratios from 1:1 to 0.2:1. In contrast, CPM incorporation was diminished impressively at the 'inflexion' point noticed at 4 × 10^3 ^input APC to T-cell ratio at 0.1:1. Furthermore, precipitous fall in CPM counts around background levels was noticed at 2 × 10^3 ^input APC to T-cell ratio at 0.04:1. Because a typical in vitro BMMC culture set at 1:1 APC to T-cell ratio, the contaminating APC, when present, fell below 10^3 ^per culture, the possibility of contaminating APC henceforth was responsible for antigen presentation in the three week old BMMC cultures, can be formally ruled out.

Thus the above data show four pertinent points for mast cells as APC: (i) pure mast cells were indeed capable of antigen presentation; (ii) pure mast cells were equally potent APC as compared to professional APC: B-cells/monocytes and DC; (iii) it is unlikely that contaminating cells at ~1% levels in the three week BMMC were responsible solely for antigen presenting capacity; (iv) it also follows that it is unlikely that the 0.38% surface FcεRI +/c-kit negative cell subset, even if they represent the basophils, can not be responsible for antigen presentation in the BMMC cultures.

**Figure 3:**
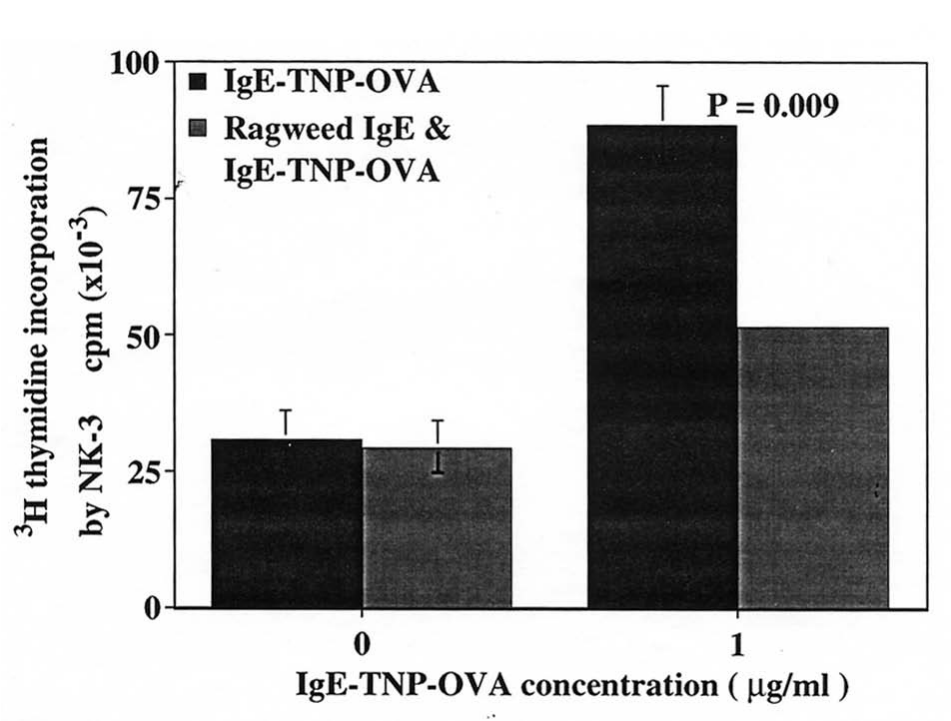
**Block of surface FcεRI-mediated antigen focusing and presentation of anti-DNP IgE/TNP-OVA by ragweed-specific IgE**. BMMC were preincubated with anti-ragweed IgE at 100 μg/ml for one hr, washed and, followed by addition of IgE/TNP-OVA complexes, and coincubated with 3Do-54.8. Levels of IL-2 in 72 hr supernatants were determined by stimulating ^3^H-thymidine incorporation and proliferation of IL-2 dependent NK-3 cells as described in Material and methods.

Augmented antigen presentation via IgE-antigen complexes, as shown in the above Fig. 2A, suggests an important role of IgE-dependent antigen presentation via FcεRI-mediated pathway. To block FcεRI receptor-mediated antigen presentation, BMMC were preincubated with anti-ragweed IgE at 100 μg/ml for one hr at 37°C prior to addition of IgE-TNP-OVA at 1 μg/ml. Fig. 3 showed that the capacity of IgE-TNP-OVA-treated mast cells to stimulate T cells was significantly reduced after treatment with anti-ragweed IgE. These results support the notion that FcεRI on BMMC plays an important role in antigen presentation.

### FcεRI^hi ^but not FcεRI^lo ^subset of three-week old BMMC is capable of MHC II-mediated antigen presentation

**Figure 4:**
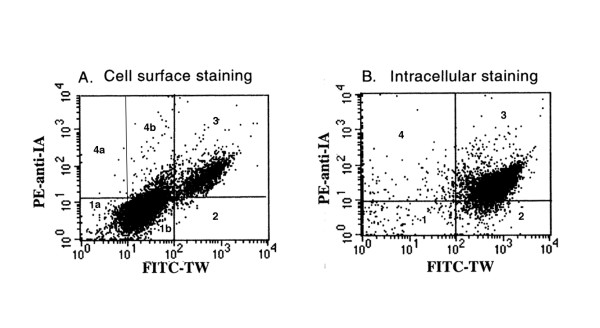
**Delineation of surface vs intracellular FcεRI/surface MHC II+ cells in three-week old BMMC**. For viable cell staining, 1 × 10^6 ^three-week old mast cells were stained with FITC-anti-FcεRIα (TW) and PE-anti-IA^d ^at 10 μg/ml and fixed with 1% paraformaldehyde. Panel A: FcεRI + cells were divided into FcεRI^lo ^(below 0.8 to the second log TW intensity), and FcεRI^hi ^cells (between second and third log TW intensity). Accordingly, quadrant #2 (.6%), #3 (35%) and #4b (13.5%) amounted to 49.1% FcεRI^hi ^cells. By contrast, quadrant 1a (9.8%) and #1b (41%) amounted to 49.8% FcεRI^lo ^cells. In total, ~99% cells were FcεRI + cells. Panel B: To determine intracellular distribution, cells were prefixed, permeabilized, and stained. Quadrant percentages were: quadrant #1 (.5%), #2 (12%), #3 (87%), and #4 (0.5%). Approximately 99% cells were FcεRI^hi ^mast cells, and 87% of these mast cells also exhibited high levels of intracellular MHC II.

The augmented antigen-presenting pathway via FcεRI prompted the hypothesis that the BMMC subset expressing high levels of FcεRI (i.e., mean fluorescence intensity of two to three logs by flow cytometry) may also express high levels of cell surface MHC II. As shown in Fig. 4A, one subset of FcεRI^hi ^subset of BMMC exhibited high levels of cell surface FcεRI and MHC II; this subset of FcεRI/MHC II high cells was delineated in quadrant 3 (35%), and 4b (13.5%) with a subtotal of 48.5% pure mast cells. Moreover, the FcεRI^lo ^subset exhibited lower but significant level of cell surface FcεRI, devoid of surface MHC II, i.e., FcεRI^lo^/MHC II(-) cells, delineated in quadrant 1a and 1b with a subtotal of 50.8%. Therefore, FcεRI^hi ^and FcεRI^lo ^constituted approximately 99% mast cells with or without expressing surface MHC II. Only 0.6% FcεRI^hi ^cells exhibited low levels of surface MHCII in quadrant 2, and very few (~0.01%) FcεRI^lo ^cells exhibited high level surface MHC II.

In striking contrast, Fig. 4B showed that despite the differential expression of cell surface FcεRI and MHC II between these two distinct subsets of mast cells, almost all BMMC exhibited high levels of intracellular FcεRI, indicating their mast cell lineage. Interestingly, in contrast to cell surface expression, nearly all mast cells exhibited intracellular MHC II, while 87% of the mast cells exhibited high levels of intracellular MHC II with 12% mast cells exhibiting lower level of MHC II. Furthermore, as anticipated, surface FcεRI^hi^/MHC II + mast cell subset, compared to surface FcεRI^lo^/MHC II(-) subset, exhibited higher levels of adhesion molecules, LFA-1α, ICAM-1, and α4 integrin and β1 integrin (data not shown).

In summary, the above data demonstrated two points: (i) ubiquitous expression of the FcεRI marker of the receptor α chain (detected by MAb TW) intracellularly ascertained the mast cell lineage of BMMC culture; (ii) distinction of mast cell subsets is made according to differential surface expression but not intracellular expression of the two employed markers, FcεRI and MHC II.

**Figure 5:**
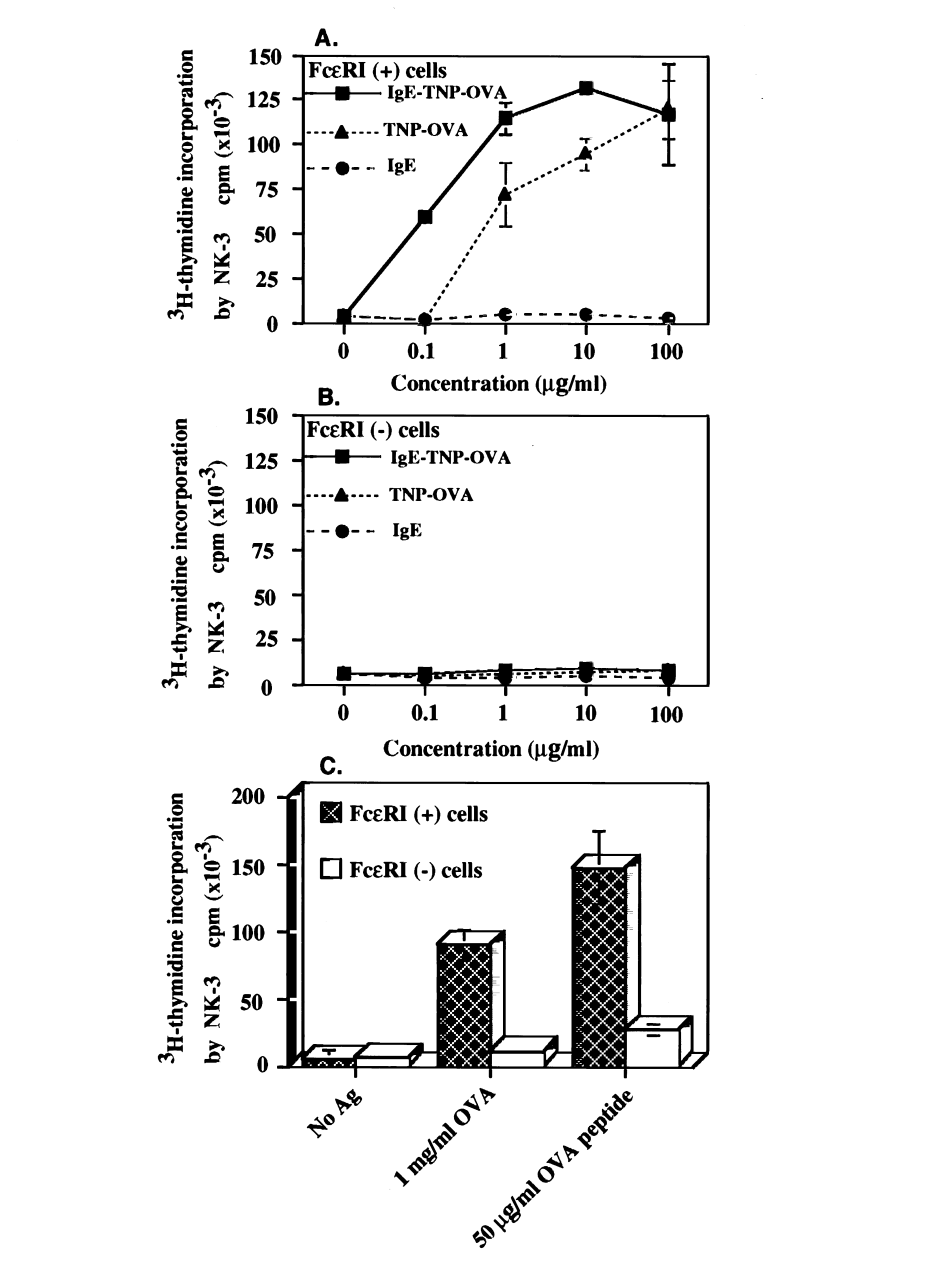
**Surface FcεRI^hi^/MHCII(+) BMMC but not surface FcεRI^lo^/MHCII(-) BMMC are capable of presenting antigens**. Three-week old BMMC were sorted into FcεRI^hi^/MHCII positive vs FcεRI^lo^/MHCII negative subsets. Each BMMC subset was then incubated with 3Do-54.8 in the presence of IgE-TNP-OVA (Panel A), TNP-OVA (Panel B) and OVA peptide (Panel C), and levels of IL-2 in 72 hr supernatants were determined by stimulating ^3^H-thymidine incorporation and proliferation of IL-2 dependent NK-3 cells as described in Material and methods.

The aforementioned observations then prompt the hypothesis that subset of FcεRI^hi ^mast cells, expressing high levels of MHC II, is competent in antigen presentation, while subset of FcεRI^lo ^mast cells, lacking cell surface MHC II, is incapable of antigen presentation. Next, BMMC were differentially sorted into the surface FcεRI^hi ^vs. the FcεRI^lo ^subset according to the surface intensity by MAb anti-FcεRIα(TW). Sorted surface FcεRI^hi ^subset of BMMC was capable of presenting all three form of antigens (Fig. 5), and as anticipated, this subset of BMMC was more efficient in presenting IgE-TNP-OVA as compared to haptenated OVA, native OVA (Fig. 5A) and peptide antigen (Fig. 5C). In contrast, purified surface FcεRI^lo ^subset, also lacking surface expression of MHCII, was incapable of presenting IgE-TNP-OVA (Fig. 5A), TNP-OVA (Fig. 5B), and OVA peptide (Fig. 5C). It is pertinent to point out that the contaminating residual APC that do not express FcεRI, should have been enriched in the FcεRI^lo ^subset. Thus, the conspicuous absence of antigen-presentation function in this BMMC subset indicates and substantiated the fact that the contaminating APC can not account for antigen presentation in BMMC/T-cell hybridoma cocultures.

**Figure 6:**
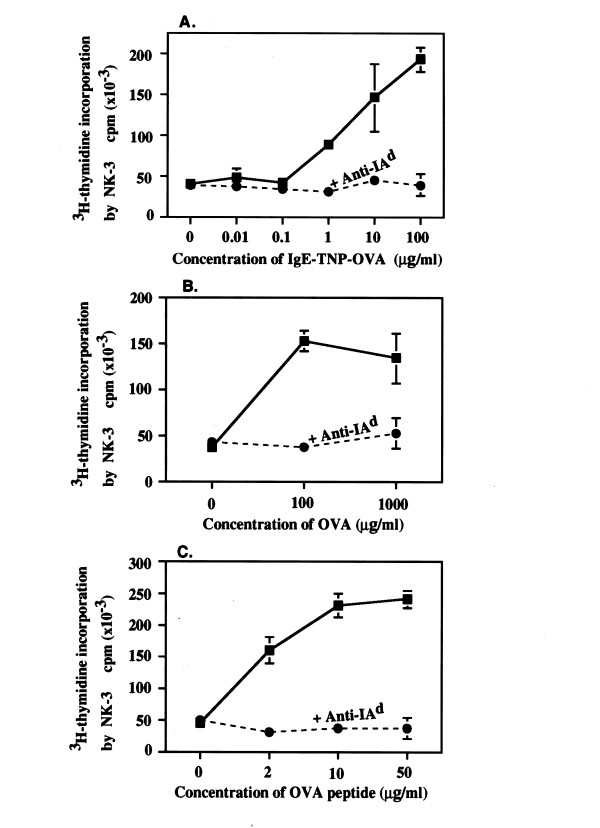
**Anti-IA^d ^potently inhibits antigen presentation by BMMC (H-2^d^)**. 10^6 ^BMMC were treated with 10 μg/ml MAb anti-IA^d ^for 30 min at 4°C. 10^5 ^treated BMMC were then incubated with 1 × 10^5 ^3Do 54.8 in the presence of IgE-TNP-OVA (Panel A), OVA (Panel B) and OVA peptide (Panel C). Levels of IL-2 in 72 hr supernatants were determined by stimulating ^3^H-thymidine incorporation and proliferation of IL-2 dependent NK-3 cells as described in Material and methods.

Consequently, we tested whether blocking surface MHC II of the FcεRI^hi ^antigen-presenting BMMC subset inhibited antigen presentation. As shown in Fig. 6, addition of MAb anti-IA^d ^profoundly inhibited IgE-dependent antigen presentation at 1 to 100 μg/ml IgE-TNP-OVA (Fig. 6A). Treatment with anti-IA^d ^also potently diminished antigen presentation of both native OVA protein and OVA peptide (Fig. 6B and Fig. 6C). In contrast, MAb against an irrelevant specificity, i.e., anti-IE^k ^(17-3-3) did not affect the antigen presenting function of BMMC (not shown).

### The antigen-presenting function of BMMC is diminished upon extended culture

Next we examined whether 4 to 6 week-old BMMC (DBA/2 mice, H-2^d^) can also present antigen Surprisingly, as shown in Table 1A, IL-3 cultured, 4-week old BMMC were incapable of presenting OVA, TNP-OVA, and the OVA peptide. The source of the IL-3 as growth factors did not make a difference, since BMMC cultured in the presence of IL-3-containing conditioned medium from D11 cells, recombinant IL-3 (100 U/ml), primed with GM-CSF all lacked antigen-presenting capacity. Under physiological conditions, stem cell factor (SCF) stimulates mast cell progenitors of various tissue origins to differentiate more toward a mature phenotype of connective tissue mast cells [5-7]. As shown in Table 1A, BMMC cultured with SCF (1 μg/ml) or a combination of SCF and IL-3 still failed to present antigen. Moreover, BMMC treated further with IL-4 (100 U/ml) [9] did not render four-week old BMMC competent in antigen presentation. In contrast, three-week old BMMC as positive control were consistently capable of presenting different forms of antigens regardless supplementation of IL-3 or SCF or priming by IL-4. Furthermore, four-week old BMMC prepared from another H-2^d ^haplotype, BALB/c mice, were likewise not capable of antigen presentation (data not shown).

**Table 1:** A - Three-week but not four-week old BMMC are capable of antigen presentation and B - Loss of APC function between three and four week old BMMC

A					
**DBA/2 (H-2^d^)**	**Culture conditions**	**Antigenic stimulation (SI)** **3Do-54.8**			**Background (CPM)**

4 week old	BMMC, GM-CSF primed	OVA	OVAp	IgE-TNP-OVA	None

	WEHI-3	0.74	1.1	.95	1,504

	D11 sup	.86	.76	.58	2,045

	Rec IL-3	.75	.80	.63	1,786

	SCF	1.3	.67	.75	1,745

	WEHI-3+SCF	.95	.87	1.0	3,164

	WEHI-3+IL-4	.79	.83	.87	2,568

3 week old	BMMC, GM-CSF primed				

	WEHI-3	7.8*	8.5*	14.4*	2,359

	SCF	10.5*	9.6*	15.2*	2,790

	WEHI-3+IL-4	8.5*	7.4*	22.3*	3,876

**B10.BR (H-2^k^)**		**Antigenic stimulation (SI)** **AD10**			**None**

4 week old	BMMC GM-CSF primed	PCC	PCCP	IgE-TNP-PCC	

	WEHI-3 sup	1.4	1.3	0.8	2,140

	+IL-4	1.2	1.3	.9	1,398

					

3 week old	BMMC, GM-CSF primed				

	WEHI-3	14.4*	9.8*	18.2*	2,575

	+IL-4	15.2*	12.1*	17.4*	2,132

**B**					

**DBA/2 (H-2^d^)**	**Culture conditions**	**Antigenic stimulation (SI)** **3Do-54.8**	**Background (CPM)**		

Days old of BMMC	GM-CSF primed, in WEHI-3 media	IgE-TNP-OVA	None		

Day 22	ibid.	10.8*	1,799		

Day 23	ibid.	12.1*	1,325		

Day 24	ibid.	14.5*	2,569		

Day 25	ibid.	12.6*	1,634		

Day 26	ibid.	3.3*	2,788		

Day 27	ibid.	0.86	2,961		

1A: BMMC (H-2^d^, or H-2^k^) were prepared by culturing bone marrow cells in the presence of different sources of IL-3 (20% supernate) or SCF (1 μg/ml) for three or four weeks with weekly decanting of adherent cells. BMMC were primed with GM-CSF (100 U/ml) for 48 hr and some cultures in addition treated with IL-4 at 100 U/ml. BMMC (H-2^k^) and PCCP-specific AD10 T-cell line were stimulated with pigeon cytochrome c (PCC), PCC peptide (PCCP) or IgE-TNP-PCC complexes at 10 μg/ml for 48 hr and IL-2 production was determined. 1B: For daily assessment of APC function from day 22 to day 27 of the fourth week, nonadherent BMMC were directly obtained from the WEHI-3 supplemented culture wares in the fourth week culture (Day 22 to Day 27) without culture media or plate change. Background CPM was determined in cocultures of differently-treated BMMC plus T cells without antigen stimulation. Stimulation index (SI) was computed as quotients of total CPM of ^3^H-thymidine uptake of the antigen-stimulated cultures over nonantigen-stimulated controls. Triplicates were set for each group of stimulation and the non-stimulated control, and the means and SE were computed and two-tailed t-test of the antigen-stimulated group compared to background CPM was performed; SE of all observations were calculated within 15% of the computed means. To simplify data, SI with p values < 0.01 was denoted as a * marks in cocultures with competent BMMC stimulation. SI of the group *without the mark *indicated a failure or loss of antigen presentation by extended cultured mast cells. L-2 secreted in the coculture supernatants was determined by stimulating proliferation and ^3^H-thymidine uptake of IL-2 dependent NK3 cell line.

We then extended the study to APC function of BMMC prepared from B10.BR mice (H-2^k^) of a different MHC haplotype. As also shown in Table 1A, four-week old BMMC failed to present PCC, TNP-PCC, and IgE-TNP-PCC to PCCP-specific AD10 T-cell line [17]. Moreover, these BMMC were not rendered competent in antigen presentation by additional IL-4 treatment [18]. The lack of antigen-presenting function by 4-6 weeks old pure BMMC was consistently observed in fifteen experiments over 12 months. Moreover, Table 1B showed that the loss of antigen-presenting function occurred precipitously in the midst between the third and fourth weeks on day 26 in the same cultures. The loss of antigen presenting function was not due to loss of adherent cell population since media were not decanted nor the cells transferred to a new dish. These observations indicate a unique temporally restricted antigen-presenting capacity inherent in bone marrow derived mast cells. In contrast, the antigen-presenting function of the purified residual professional APC were fully functional throughout the entire 3-week 3 to 6-week cultures (not shown).

## Discussion

Herein, the study shows that highly pure mast cells from three week old, IL-3-containing bone marrow cell cultures indeed present antigens after depletion of ~1% contaminating APC. This study also provides the novel observations: (i) two subsets of BMMC are delineated; one subset expressing both surface high levels of FcεRI and high surface MHC II, while another expressing low levels of surface FcεRI with non-detectable levels of surface MHC II. Nevertheless, the intracellular expression of both biomarkers are equivalent in all BMMC; (ii) the purified FcεRI^hi ^BMMC subset that express high levels of surface MHC II, is competent in antigen presentation, while the FcεRI^lo ^subset can not present antigens. Since FcεRI^lo ^was presumably enriched even 2-fold for the putative contaminating APC, its conspicuous lack of capacity for antigen presentation strongly argues against the role of contaminating professional APC for stimulating T-cell hybridomas in BMMC/T-cell cocultures; (iii) augmented IgE-dependent antigen presentation pathway in the FcεRI^hi ^subset is inhibited by IgE of a different specificity, and the APC function is blocked by anti-MHC II antibodies; (iv) the APC function is also temporarily restricted to the three weeks old BMMC, and is irrevocably lost in further extended cultures.

Previously, Mecheri and colleagues showed BMMC cultured in ConA-conditioned media or two-way mixed lymphocyte reaction (MLR) supernatant present antigens to stimulate CD4 + helper T cells [9,19]. However, there has been continual debate concerning the purity of BMMC and the role of possible contaminating APC. Our previous study in collaboration with Stevens, Austen and colleagues [14,20] also showed three-week old BMMC grown in WEHI-3-conditioned media were authentic mast cells by EM studies, granular content, as well as release of histamine, PDG2 and leukotrienes via IgE-mediated cell activation. The present study also confirms the granularity and histamine content in almost all the cells in three-week old BMMC cultures. Approximately 98.7% BMMC expressed high density of c-kit and high vs low density of surface FcεRI (Fig. 1g and Fig. 4A), and nearly all these BMMC uniformly exhibited high levels of FcεRIα in the cytosol (Fig. 4B).

Basophils, present in less than 1% circulating leukocytes were recently shown to play a critical role in innate immunity and especially in orchestrating Th2 commitment [21]. Although the present culture system did not support basophils, a possible low contamination (~0.38%) can not be excluded [16]. Since antibodies to CD200R3 (Ba91 and Ba103) directly activate degranulation of basophils as well as mast cells in vitro [21-24], these antibodies may not be directly employed for depleting minor contaminating basophils in BMMC cultures without a concomitant modulatory effect on antigen presentation by mast cells. On the other hand, these antibodies appeared to eliminate preferentially basophils in vivo. Thus, future studies may be conducted in vivo for evaluating antigen-presenting function of adoptive transferred BMMC, while preferentially depleting basophils in situ in an appropriate animal model deficient in mast cells as well as all the other professional APC cell types. This study also relies on supply of a large quantity of antibodies produced by hybridoma clones, available to the research laboratory [21-24].

Herein, we formally showed that physically purified, surface FcεRI^hi ^but not the cell surface FcεRI^lo ^(or null) BMMC subset present antigens. Noteworthily, these sorted surface FcεRI^lo ^BMMC are also authentic mast cells according to the abundant expression of the lineage-specific FcεRIα marker in the cytosol [25,26] (Fig. 4B) and surface c-kit (Fig. 1g) [16]. Residual contaminating professional APC did not play a role in these BMMC cultures for three reasons: (i) three week old BMMC depleted of contaminating APC were fully capable of antigen presentation (Fig. 2A-C); (ii) surface FcεRI^lo ^BMMC, presumably enriched for the putative professional APC, were not capable of antigen presentation; (iii) purified professional APC added to T-cells at a simulated ratio of BMMC/T-cell cultures, i.e., 10^3 ^contaminating APC to 10^5 ^T-cells, failed to stimulate IL-2 production by T-cell hybridomas (Fig. 2F).

Augmented presentation of antigen-IgE complexes can be mediated via the low affinity FcεRII pathway [27]. Herein augmented presentation of antigen-IgE complexes was mediated via the high affinity FcεRI pathway, blocked by preincubation with monomeric ragweed-specific IgE. Mast cells are increasingly known for downregulating a variety of immune effector functions in skin graft rejection, protozoan infection and UVB-induced contact dermatitis [28-30]. In addition to the straightforward receptor blockade mechanism, the diminished antigen presentation may be in part explained by suppression of APC function due to receptor interactions with monomeric ragweed-specific IgE in contrast to multivalent interactions with TNP-OVA-IgE.

It is not known why low levels of cell surface FcεRIα correlated with lack of MHC II cell surface expression (Fig. 4A). MIIC-like compartments were noted intracellularly in mast cells [31]. It is possible that there exists a special mechanism, co-transporting intracellular pools of FcεRI and peptides/MHC II in the MIIC-like compartment to the cell surface [31]. Alternatively, the lack of cell surface expression of the alpha subunit of FcεRI may be due to lack of intracellular expression of β/γ subunits [25,26], while surface MHC II expression depends on vesicular transport of MIIC compartments to cell surface during BMMC differentiation. Since exosomes secreted by RBL-2H3 can arm professional antigen presenting cells [31,32], it remains possible that the FcεRI^lo ^mast cell subset may indirectly contribute to antigen presentation via cross-presentation of secreted exosomes by other professional APC [32,33].

The mechanisms for sudden collapse of APC function of BMMC on day 26 BMMC and further extended cell cultures of 4-6 week in vitro, are not unknown. Many possibilities exist. It is interesting to speculate that contaminating basophils and/or professional APC from ~400 cells to 1,000 cells in the APC/T-cell cocultures may play an indirect role in modulating competence of mast cells in antigen presentation; and starvation of one or both cell types in extended cultures may account for the temporally restricted nature of mast cells as a facultative APC [28,34]. The elucidation of cellular and molecular mechanisms underlying the spatiotemporally restricted nature of pure mast cells as APC, remains a future challenge for basic mast cell research and its clinical application.

## Conclusions

Mast cells are increasingly known for modulating a variety of immune effector functions in skin graft rejection, protozoan infection and UVB-induced contact dermatitis [28-30]. The observation herein provides an additional link for integrating inflammatory responses with spatiotemporally restricted antigen presenting function via IgE-dependent antigen presentation pathway in this unique FcεRI^hi ^/MHC II^+ ^BMMC subset. Coupling antigen presentation with secretory function of mast cells via the interplay of cell surface FcεRI and MHC II can contribute to important pathophysiological responses in both innate and acquired immunity of the host [24,28,34,35].

## Abbreviations

APC: Antigen-presenting cells; BMMC: Bone marrow-derived mast cells; DC: Dendritic cells; FcεRI: Type I high affinity IgE Fc receptor.

## Authors' contributions

JG for designing, executing the overall experiments, and contributing to the draft; ICW for determining purity of mast cells; LS for validating APC function and data analysis; MC for providing and guiding PCCP presentation system; NSY for data analysis and editing; FTL for conception, designing, supervising and writing the manuscript; SSC for conception, designing, supervising and writing the manuscript. All authors read and approved the manuscript.
